# SNAD: sequence name annotation-based designer

**DOI:** 10.1186/1471-2105-10-251

**Published:** 2009-08-14

**Authors:** Igor A Sidorov, Denis A Reshetov, Alexander E Gorbalenya

**Affiliations:** 1 Molecular Virology Laboratory, Department of Medical Microbiology, Center of Infectious Diseases, Leiden University Medical Center, PO Box 9600, E4-P, 2300 RC Leiden, Netherlands; 2 Faculty of Bioengineering and Bioinformatics, Moscow State University, 119899 Moscow, Russia

## Abstract

**Background:**

A growing diversity of biological data is tagged with unique identifiers (UIDs) associated with polynucleotides and proteins to ensure efficient computer-mediated data storage, maintenance, and processing. These identifiers, which are not informative for most people, are often substituted by biologically meaningful names in various presentations to facilitate utilization and dissemination of sequence-based knowledge. This substitution is commonly done manually that may be a tedious exercise prone to mistakes and omissions.

**Results:**

Here we introduce SNAD (Sequence Name Annotation-based Designer) that mediates automatic conversion of sequence UIDs (associated with multiple alignment or phylogenetic tree, or supplied as plain text list) into biologically meaningful names and acronyms. This conversion is directed by precompiled or user-defined templates that exploit wealth of annotation available in cognate entries of external databases. Using examples, we demonstrate how this tool can be used to generate names for practical purposes, particularly in virology.

**Conclusion:**

A tool for controllable annotation-based conversion of sequence UIDs into biologically meaningful names and acronyms has been developed and placed into service, fostering links between quality of sequence annotation, and efficiency of communication and knowledge dissemination among researchers.

## Background

Identifiers (ID) composed of alphanumeric and other symbols are commonly used for tagging protein and nucleotide sequences and associated annotation in GenBank [1], UniProt [2], and other sources that either use these sequences or deal with molecular properties of associated biological entities (Table 1). For instance in UniProt, the primary accession number is the sole UID. In GenBank three fields, locus, accession and version, carry four different IDs, but only gi number (known also as primary ID) and accession number combined with version form unique IDs (UIDs). Each UID is assigned to a single entry. In contrast, a particular non-unique ID may be found in numerous entries. The UIDs ensure efficient computer-mediated data storage, maintenance and processing. To facilitate data mapping and transfer between different databases, in which ID inter-conversion plays important part, several specialized resources including GoMiner [3], ErmineJ [4], SOURCE [5], RESOURCERER [6]; IDconverter and IDClight [7], and DAVID [8] have been developed.

**Table 1:** Unique and non-unique identifiers in databases used by SNAD

**Database**	**Identifier**	**Uniqueness**	**Example**
GenBank^1^	gi number, primary ID	+	1234567
	version number = accession.version	+^9^	AY123456.1
	accession number	-	AY123456, NC_006558, NP_000483
	locus/sequence name	-	MTVCG, SCU49845
UniProt^2^	accession number	+	Q10AA9, P47123, A2BC19
	entry name	-	INSR_HUMAN
EMBL^3,4,5^	accession number	+	X64011, M10051, J00231, AJ000001, AY123456
	entry name	-	HSINSR, BUM
SeqHound^6,7^	gi number	+	1234567
	accession number	-	AY123456, NC_006558, NP_000483
	locus/sequence name	-	BTACHRE
EnsEMBL Gene/Transcript for Human^8^	gene ID, transcript ID	+	ENSG00000133103, ENST00000222982

1)

2)

3) [28]

4)

5)

6) [29]

7)

8) Gene and Transcript part of EnsEMBL,

9) Some replaced or removed records in GenBank have no accession number but they can be retrieved from GenBank with the primary ID.

Sequence IDs are used for tagging sequences also in multiple sequence alignments and sequence-based phylogenetic trees in various presentations including publications [9-12]). Notwithstanding UniProt/SwissProt [2] entry names (Table 1), these machine-readable IDs are not very informative in human communications. Consequently, sequence IDs are commonly replaced by biologically meaningful names and acronyms, e.g. species names in trees and gene/protein names in alignments. When a large-scale dataset is to be processed, manual replacement of IDs with names could be a tedious task prone to mistakes and omissions.

By some considerations, the ID-to-name conversion for viral sequences is both of particular relevance and of considerable complexity. First, a large body of results in virus research can be linked to underlying sequences, owing to relatively small number of known viruses and comparatively small sizes of their extensively characterized genomes. Second, the virus naming, at the species level and below, is not governed by either the binomial Latin name convention traditional for cellular organisms or another broadly accepted nomenclature [13] that complicates communication in virology. For species, naming viruses is under auspices of the International Committee of Virus Taxonomy (ICTV) [14]. At the isolate and strain level, it is left up to individuals or groups of researchers to decide how to proceed [15,16]. Researchers studying few economically most important viruses developed nomenclatures restricted for these viruses [17-22]. In contrast, each decision about naming that concerns a majority of viruses is an *ad hoc *and, often, lengthy process. Likewise, naming viruses with artificially designed genomes, which are not product of natural evolution [23], is formally outside of the ICTV authority and completely unregulated.

The problem of ID-to-name conversion has been partially addressed in the NEXUS alignment format [24], which has a reserved part (block) for converting names in a predefined manner, and in the stand-alone tool Phyutility [25] that allows combining a limited number of GenBank characteristics with a custom separator to form sequence names. Here we introduce a dedicated web tool dubbed Sequence Name Annotation-based Designer (SNAD) that provides a versatile facility with user-friendly interface for automatic conversion of sequence UIDs into biologically informative names and acronyms in controllable manner using annotation associated with sequences. Several examples are provided to illustrate how SNAD can be used to generate names for practical purposes, particularly in virology.

## Implementation

SNAD (ver. 1.6) is accessible at . SNAD code is written in Perl (BioPerl for dynamic retrieval of entries from databases, CGI for interfacing Perl code with web server, original Perl code for analysis of sequence objects retrieved from databases, and conversion of sequence annotation into complex names, part of application programming interface – API), JavaScript (graphical user interface for uploading data, designing format of name conversion and representing results), Java (part of API) and HTML for SNAD web site pages (see  where a detailed description of all SNAD options and parameters can be found). SNAD can be used as a web server with APIs in Perl and Java available on SNAD web site. SNAD recognizes input as alignment or tree with the use of BioPerl drivers (Bio::TreeIO and Bio::AlignIO). If the input is not recognized as alignment or tree, SNAD processes it as a list of identifiers separated by one or more of the following symbols: space, new line, tabulator, or comma. To map IDs on database entries, Bio::DB::(GenBank, SwissProt, EMBL or SeqHound) drivers and Bio::EnsEMBL::DBSQL::DBAdaptor are used. SNAD uses standard BioPerl functions from Bio::Seq for extracting features/annotation from the database entries (GenBank/RefSeq/GenPept, UniProt, EMBL, SeqHound) or functions from database programming interface (EnsEMBL). Detailed description of all available functions is presented in "Introduction" page of the web site. SNAD code is available for downloading by non-commercial users per request sent to SNAD@lumc.nl.

## Results and discussion

**Figure 1:**
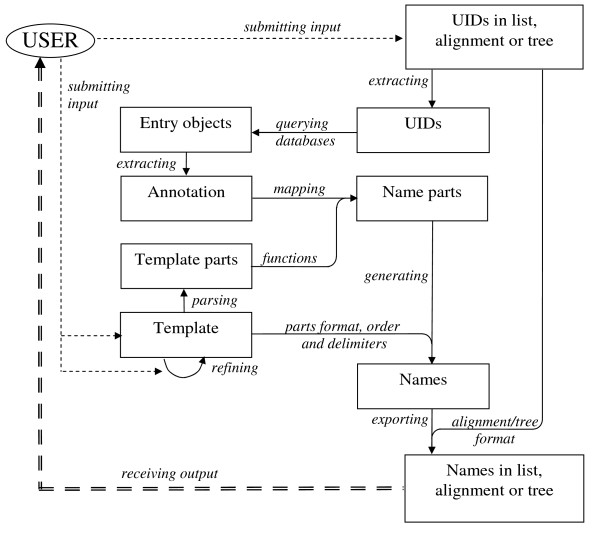
**SNAD dataflow**. User submits sequence UIDs and defines a format of conversion by choosing a template. SNAD analyzes the input, extracts UIDs from it and queries a user-defined database to locate cognate entries. The next step is extracting annotation from the entries and parsing the user-selected template to generate separate parts. They are mapped onto annotation to produce name parts, which are combined into names according to the template. Finally, SNAD substitutes the submitted UIDs in list, alignment, or tree with the designed names and returns results to the user.

The diagram of SNAD dataflow is shown in Figure 1. The user submits sequence IDs, defines a format of conversion, and selects a database to be searched. Currently available databases include GenBank/RefSeq/GenPept, UniProt, EMBL, SeqHound and EnsEMBL (Gene and Transcripts divisions for Human sequences). SNAD extracts IDs from the input and queries the database with these IDs to locate associated annotation. After retrieving annotation, SNAD designs names according to a name template that defines name structure and annotation-based content for submitted entries. The final step of the SNAD-mediated processing is substitution of submitted IDs in the input with the designed names to return results to the user.

**Figure 2:**
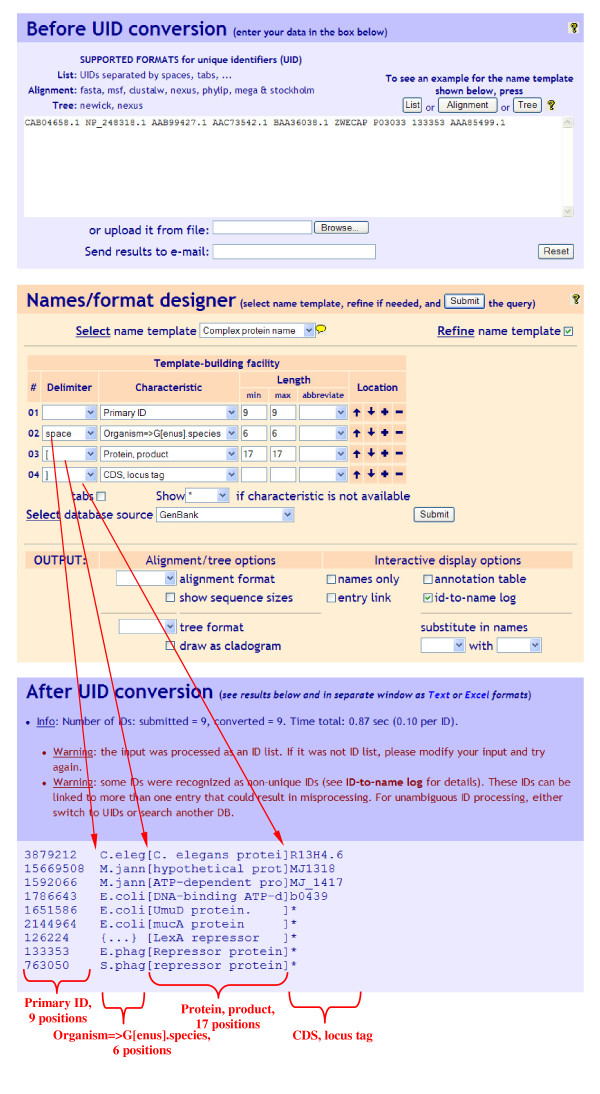
**Unique IDs conversion with SNAD**. Nine IDs from GenBank (section "Before UID conversion") are converted using a pre-compiled four-characteristic template "Complex protein name" that includes the following GenBank characteristics: "Primary ID" (gi number); organism name formatted as "G [enus].species"; protein product and gene locus tag. Three delimiters are used: space, " [" and "]", and none of characteristics is abbreviated. First three characteristics have their size limited to: 9, 6, and 17 symbols, respectively. Results of the conversion are shown in "After UID conversion" section. Each arrow links a delimiter to a part of converted names. {...} indicates that more than one organism name characteristic is found for a submitted ID (see [GenBank/Protein: 126224], features source/organism).

The SNAD web site has six pages including a detailed guide to SNAD with examples ("Introduction"), program execution page ("Execution"), guided access to Java and Perl APIs ("APIs"), frequently asked questions page ("FAQs"), list of the SNAD versions ("History"), and "Reference" page. SNAD execution page has three vertically arranged sections: "Before unique ID (UID) conversion" for uploading input, "Names/format designer" for choosing and refining pre-compiled name templates, and "After UID conversion" for accessing output (Figure 2).

The input can be either copy-pasted or uploaded from a file. SNAD accepts IDs from five databases (Table 1) including GenBank (gi or accession number) and UniProt (accession number or entry name) that are automatically recognized in and extracted from the input data prepared in several formats: as part of tree ('newick' and 'nexus') and alignment ('fasta', 'msf', 'clustalw', 'nexus', 'phylip', 'mega' and 'stockholm') or supplied as a plain list (Figure 2). Examples of IDs, unique and non-unique, in alignment, tree, and plain list are available for testing purposes. SNAD may accept input with non-unique IDs, e.g. GenBank accession number or locus name, that are most popular among researchers. Since these IDs may be associated with multiple entries, SNAD must decide which entry is to be used to retrieve annotation. The adopted approach is to use the most recent entry that might be not the one the user aimed at to retrieve annotation. The user may request automatically generated ID-to-name log (see below) for checking the quality of the conversion. To ensure unequivocal ID processing the user must use UIDs in input. UIDs from one database can be used to query another non-cognate database in which it may be not recognized at all or recognized as non-unique IDs (Table 2). For instance, UIDs from UniProt (primary accession numbers) are recognized as non-unique IDs in GenBank (accession numbers) while all UIDs from GenBank (gi or accession.version) are not recognized as valid IDs in UniProt.

**Table 2:** Cross-database recognition of UIDs in databases by SNAD*

	**Source DB**				
	
**Target DB**	GenBank	UniProt	EMBL	SeqHound	EnsEMBL
GenBank	UID	NUID	NUID	UID	NR
UniProt	NR	UID	NR	NR	NR
EMBL	NR	NR	UID	NR	NR
SeqHound	UID**	NR	NUID	UID	NR
EnsEMBL	NR	NR	NR	NR	UID

*With the use of standard BioPerl drivers for GenBank, UniProt, EMBL and SeqHound  and API drivers from EnsEMBL web site , UIDs from source DB can be used for searching in target DB. In some target DBs they are recognized as UID, in others as non-unique IDs (NUID) or not recognized as identifiers (NR). Although same five DBs make the source and target DB lists, the table is *not *symmetrical.

**, only gi's from GenBank are recognized as UID in SeqHound.

The user has a choice of reading SNAD output either on the "Execution" web page, or at a separate web page in plain text mode, or receiving it via e-mail. For tree input, SNAD generates, additionally to the standard output, a respective tree drawn to facilitate visual inspection of the generated names in graphic format. The web page output can be enhanced with links to cognate entries accessible through the generated names.

There are two ways of defining format of the UID conversion: using either precompiled or user-defined name templates. The user is currently provided with several precompiled templates for name generation, which can be used "as is" or after refinement (see below). Each template is accompanied with examples of input to illustrate its usage. "Entry name" template can be useful for identifying misspelled identifiers, as well as for comparative inspection of basic information about the entries in UniProt. Two other templates ("Gene" and "Species") can be used for converting UIDs into gene and species names, respectively, as they are specified in GenBank. The "Complex species name" template combines full species name with primary ID (gi) that are neatly separated. The "Complex protein name" includes primary ID, condensed species name, protein description and CDS, and locus tag, in a size restricted manner to facilitate reading a complex name structure. These templates are suited for producing names of sequences originated from a wide range of organisms. "Acronym" template converts GenBank organism name annotation into acronym. Three templates were designed specifically for viruses to address specifics of this group for which the naming remains a matter of debate [15,17]. For several medically important viruses with large sampling from the natural diversity, e.g. influenza and noroviruses [18,22], names conventions were accepted by the field to include reference to serotype, host species, country of isolation, strain or isolate name, collection date and, for influenza viruses, subtype. These names are part of the GenBank entries where from they can be readily extracted. To demonstrate that similar names can be also generated on-the-fly from two components, using the isolate/strain and serotype fields in respective entries, a separate "Influenza virus name" template is provided. We extended this approach by designing two other virus templates, "Complex virus name I" and "Complex virus name II", that could generate names with complex structure for majority of other viruses for which no consensus on the naming has been reached. These templates combine information from up to six separate entry fields, each keeping an elementary record e.g. about date or place of virus isolation. "Complex virus name II" additionally includes reference to protein function. These templates could help researchers studying many viruses, e.g. coronaviruses and picornaviruses [15] communicate their sequence-based data in a name format similar to that accepted for influenza virus. SNAD-mediated solution to the generation of complex virus names reinforces the role of diverse fields in sequence entries as the primary source of information. If these fields are completed with records as expected, virus names can be readily generated under different and evolving conventions. Thus, SNAD provides a tool for exploration of different name arrangements that could facilitate the development of conventions for naming different viruses by the virology community.

SNAD users can initiate refining of any of the provided templates (by using "Refine name template" option) or generate *de novo *templates by choosing "User defined" template that automatically activates the refining mode. In both instances, the advanced template-building facility becomes available for the user. It empowers the user with fine control over the output name format, including the number of independently designed parts of the name, characteristic of each part, parts order, size of each part, and type of delimiter preceding each part (Figure 2). Design of each part in the name is controlled by a separate row. The name parts – from left to right – are defined by respective rows in the template-building facility from top to bottom. Rows can be moved up and down or deleted and inserted with a respective effect on the name structure and content. For retrieving sequence characteristics from database entries SNAD uses BioPerl scheme with internally controlled vocabulary of annotation [26]. Information from two or more annotation fields can also be combined in a single characteristic in a template. For instance, for virus name templates, a combination "strain OR isolate" may be used to form a characteristic. For name designing, the user can choose from a dropdown menu of sequence characteristics and features that were derived from available databases. These characteristics can be used separately or in combination to design a name format that can combine, for instance, unique reference to the original database with a concise biological description of the entry (see Figure 2 for an example). For characteristics which values are described with several words, first letters of these words can be combined using characteristic's "abbreviate" option to produce acronyms. If a selected characteristic is not available in annotation for a sequence, it is replaced in the sequence name with a symbol that the user selected from a separate drop-down menu. Due to gaps in annotation, names designed with the templates may not be complete for every entry.

SNAD has a menu that controls output display interactively. By default, output includes ID-to-name log in which selected information about generated names is detailed in table format that can be downloaded as an Excel file. This table can be useful for controlling the name generation procedure and preparing legends to accompany an alignment or a phylogenetic tree in publication. The user can also extract IDs from submitted alignment or tree by activating "names only" option, or retrieve annotation from cognate entries in table format. In the current implementation of SNAD, the most time-consuming stage is data retrieval from databases. For large queries and complex name formats, also using API, it is therefore advisable to start with less than 10 sequences to design a format to be used for the full query. Queries containing up to 500 IDs and including sequence alignments up to 50000 characters can be processed through SNAD web page. For queries containing more than 500 IDs, the user is advised to use one of the SNAD APIs.

The availability of description for characteristics in a particular set of entries from databases is a major factor determining how well names designed with SNAD match expectation. The amount and accuracy of annotation in sequence databases vary markedly between entries that has been a major concern for the bioinformatics community continuously working toward improving this critical aspect of sequence databases [7,8,27]. Involving other researchers in this effort, especially those who submit original sequence annotation in the first place, could accelerate the process. We hope that through using SNAD service, researchers could see immediate benefits from better annotation for their research that may create impetus for contributing quality annotation to public databases. To improve the user experience with SNAD, we plan to expand the diversity of databases and templates upon future upgrades of SNAD. Any template that the users designed and considered useful could be included in the future SNAD releases.

## Conclusion

A tool for controllable annotation-based conversion of sequence UIDs into biologically meaningful names and acronyms has been developed and placed into service, fostering links between quality of sequence annotation, and efficiency of communication and knowledge dissemination among researchers.

## Availability and requirements

Project name: SNAD, Sequence Name Annotation-based Designer. Project home page: . Operating system(s): web service, platform independent. Programming languages: Java, JavaScript, Perl, HTML. Restrictions to use SNAD web site by non-academics: none.

## Authors' contributions

AEG conceived SNAD; IAS, DAR, and AEG developed SNAD design and performed testing; DAR and IAS carried out programming; IAS and AEG wrote the manuscript. All authors read and approved the final version of the manuscript.
